# Concurrent Imaging and Clinical Study of the Efficacy of Hyaluronic Acid Injection for Knee Osteoarthritis: A Synovial Membrane Investigation with Ultrasound Imaging

**DOI:** 10.3390/ph16081186

**Published:** 2023-08-21

**Authors:** Chien-Chih Wang, Tsung-Ming Hu, Chien-Lung Chen, Chung-Chih Hong, Yu-Hui Chang, Chung-Lan Kao

**Affiliations:** 1 Department of Physical Medicine and Rehabilitation, Taipei Veterans General Hospital Yuli Branch, Hualien 981002, Taiwan; ccwang10@ym.edu.tw; 2 Department of Physical Medicine and Rehabilitation, School of Medicine, National Yang Ming Chiao Tung University, Taipei 11221, Taiwan; 3 Department of Psychiatry, Taipei Veterans General Hospital Yuli Branch, Hualien 981002, Taiwan; tsungming.hu@gmail.com; 4 Department of Future Studies and LOHAS Industry, Fo Guang University, Yilan 262307, Taiwan; 5 Taipei Hospital, Ministry of Health and Welfare, New Taipei City 24213, Taiwan; cclong7182@gmail.com; 6 Institute of Hospital and Health Care Administration, National Yang Ming Chiao Tung University, Taipei 11221, Taiwan; 7 Tri-Service General Hospital Songshan Branch, Taipei 10508, Taiwan; iamwholeworld@yahoo.com.tw; 8 Department of Nursing, Taipei Veterans General Hospital, Taipei 11220, Taiwan; dingdingchang0212@gmail.com; 9 Department of Physical Medicine and Rehabilitation, Taipei Veterans General Hospital, Section 2, Shi-Pai Road, Taipei 11220, Taiwan; 10 Center for Intelligent Drug Systems and Smart Bio-Devices (IDS2B), National Chiao Tung University, Hsinchu 30010, Taiwan; 11 Institute of Clinical Medicine, National Yang Ming Chiao Tung University, Taipei 112304, Taiwan

**Keywords:** ultrasound, osteoarthritis, knee pain, synovitis, hyaluronic acid

## Abstract

We investigated whether hyaluronic acid (HA) injections can ameliorate ultrasound-detected synovitis in knee osteoarthritis (OA). We recruited 103 patients with symptomatic knee OA and ultrasound-detected synovitis and performed two ultrasound-guided fluid drainage procedures, followed by the administration of a low-molecular-weight HA injection (2.5 mL) in the subpatellar bursa, at a 2-week interval. Knee ultrasound imaging evaluations were performed before injection (baseline) and at 1 and 6 months after the second injection and included the measurements of synovial vascularity by using color Doppler ultrasound, synovial fluid depth over the suprapatellar bursa (SF), and synovial hypertrophy (SH). Initial clinical assessments included a visual analog scale (VAS) and the Western Ontario and McMaster Universities Osteoarthritis Index (WOMAC). VAS scores decreased significantly at both 1-month and 6-month evaluations (*p* < 0.001). WOMAC scores also significantly decreased at 1 month (*p* < 0.001), but not at 6 months (*p* = 0.23). The ultrasound parameters did not significantly change, except color Doppler grading, which tended to decrease at the 6-month evaluation (*p* = 0.059). Our findings revealed that two ultrasound-guided HA injections following fluid drainage improved pain and knee function but did not considerably influence imaging-detected synovitis in patients with knee OA.

## 1. Introduction

Osteoarthritis (OA) is a leading cause of disability in older adults and can affect all axial joints, including the spine, and appendicular peripheral joints. Knee OA causes a progressive decline in ambulation, considerably impairing activities of daily living [1]. More than 20% of all individuals over the age of 40 have pain and disability related to knee OA [2]. These individuals also experience considerably increased direct and indirect costs, including higher medical costs and a loss of productivity [3,4].

Knee OA involves all of the tissues of the knee joint and is characterized by cartilage and meniscal degeneration, subchondral bone remodeling, synovial membrane inflammation, infrapatellar fat pad inflammation, and fibrosis [5,6]. Low-grade inflammation, rather than wear and tear, is believed to be the primary pathophysiological mechanism [6]. The inflammatory event is initiated by meniscal injury or degeneration, causing meniscal debris to enter the joint, which triggers an immune reaction. The subsequent release of inflammatory cytokines into the joint induces chondrocyte hypertrophy and death, and synovial hypertrophy, as well as increases peripheral vascularity, thereby exacerbating the immune response; this becomes a vicious cycle [7]. Furthermore, synovitis causes accelerated chondrocyte death because it is a source of nutrient supply and maintains homeostasis of the soft tissues around the joint [7]. It is also a major source of pain due to the dense nociceptive innervation of the synovium [8]. Synovitis is significantly associated with knee pain and difficulty in ambulation [9].

Chronic conditions involving low-grade inflammation, such as diabetes and obesity, also contribute to and exacerbate the intra-articular inflammatory cascade in knee OA, due to insulin resistance and chronic hyperglycemia [10]. These pathogenetic mechanisms cause oxidative stress and the release of proinflammatory cytokines and advanced glycation end products, resulting in damage to the joint [10,11]. Several pharmaceuticals, including glucosamine and chondroitin, have been developed to decrease systemic inflammation and reduce pain; however, the evidence remains limited [12,13,14]. Nonsteroidal anti-inflammatory drugs can reduce pain; however, they are not appropriate for patients with comorbidities or for long-term use because they are associated with a slight increase in the risk of adverse events [15]. Intra-articular therapy offers advantages in the management of osteoarthritis in terms of higher efficacy, more effective symptom control, and fewer adverse effects.

HA (Hyaluronic acid) is one of the most widely used intra-articular regimens for knee OA [16] because of its efficacy in terms of joint lubrication, anti-inflammation effects, and analgesia [17,18]. HA is a polymer of disaccharides, which are composed of D-glucuronic acid and *N*-acetyl-D-glucosamine. Its function depends on the rheological properties of molecular weights. HA is classified into high-molecular-weight (HWM) HA (molecular weight: >6.0 million Da) and low-molecular-weight (LMW) HA (molecular weight: 0.5–3.6 million Da) [17]; each elicits a different cell response after binding to the target protein CD44 [19]. HMW HA may have better shock absorption and longer analgesic effect, whereas LMW HA may be more efficacious in reducing synovial inflammation [20]. We previously reported that LMW HA successfully alleviated active synovitis in patients with rheumatoid arthritis (RA) in the ankle [21].

Intra-articular HA injections also have antioxidant and antiapoptotic functions in knee OA [22], thus theoretically making them superior to intra-articular steroid injections [23,24]. Hyaluronic acid (HA) injection remains one of the most common alternative therapies for treating OA [18]. A clinical study revealed that HA injection for knees with OA markedly delayed the time to total knee replacement compared with placebo injections [19]. The main therapeutic effects are lubrication, decreased inflammation, pain reduction, and potential tissue repair [20]. An arthroscopic study indicated that HA modified the inflamed synovial membrane toward the normal synoviumin knee OA [25]. However, studies have provided inconsistent results. HA has also been reported to have limited efficacy and to cause only slight pain reduction in knee OA compared with placebo [26,27]; therefore, it is still not recommended in treatment by some rheumatology and orthopedic societies causing HA to be recommended *against* by certain rheumatology and orthopedic societies [28,29,30].

At present, the most common manner of evaluating OA in the knee is Kellgren–Lawrence (KL) grades based on knee X-rays, with such grades reflecting the presence of osteophyte formation and joint space narrowing [31]. However, X-rays can only be used to assess the bony part of the knee. Musculoskeletal ultrasound imaging is the most sensitive tool for evaluating synovitis [32]. Musculoskeletal ultrasound imaging offers several advantages for the real-time and dynamic evaluation of synovial conditions, such as vascularity, effusion, cartilage erosion, and soft tissue injury severity [33]. It is the gold standard for diagnosing synovitis in RA [34]. We previously reported that an ultrasound can help in determining the effects of HA injection on ankle synovitis in RA and in detecting hyperemia and synovial hypertrophy [21]. Given that knee synovitis plays a major role in OA progression, ultrasound-detected knee synovitis is strongly associated with symptoms of knee synovitis [9,35]. Although a knee ultrasound has limited utility in the evaluation of deep structures and bursas, the suprapatellar bursa can serve as the representative bursa for detecting knee inflammation; ultrasound detection of suprapatellar effusion and the associated synovial vascularity are closely linked to the symptoms of knee synovitis [9,36].

The leading cause of the poor effects of HA therapy is likely the high severity of knee OA or synovitis, which is determined on the basis of KL grades and ultrasound findings [35]. However, how HA therapy actually affects synovitis in knee OA remains unclear. We hypothesized that HA injections might ameliorate the ultrasound-detected signs of synovitis, including synovial fluid accumulation, increased vascularity, and synovial hypertrophy, and can improve clinical outcomes. In the current study, we investigated whether the clinical improvement of the OA knee under HA therapy is associated with the ultrasound-detected amelioration of synovitis. We chose LMW HA because of its good efficacy in reducing both global and functional pain [21,37] and better synovial penetration than HMW HA [17,21].

## 2. Results

### 2.1. Patient Characteristics

We initially recruited 115 patients, of whom 12 did not complete the evaluation for personal reasons. Finally, 103 patients were included in the final analysis (Table 1). Significant clinical improvement was observed after HA injections. Compared with baseline values, VAS scores were significantly lower at both the 1-month (*p* < 0.001) and 6-month evaluations (*p* < 0.001), whereas WOMAC scores were significantly lower at the 1-month evaluation (*p* < 0.001), but not at the 6-month evaluation (*p* = 0.25; Table 2).

### 2.2. Ultrasound Synovial Outcome

In the ultrasound evaluation, significant changes were not observed in SF, which changed from 0.49 cm at baseline to 0.50 cm at the 1-month evaluation (*p* = 0.49) but decreased slightly to 0.48 cm (*p* = 0.118) at the 6-month evaluation. Synovial vascularity non-significantly decreased from 0.66 (0.52–0.79) at baseline to 0.63 at the 1-month evaluation (*p* = 0.102) and 0.58 at the 6-month evaluation (*p* = 0.06). The responder rate indicated that a total of 7.7% of the patients with grade 1 color Doppler at baseline had grade 0 after therapy, whereas none of the patients with grade 2 at baseline exhibited any change (Table 2). SH remained unchanged at both of the 1-month and 6-month evaluations (*p* = 0.918 and 0.446, respectively; Table 3).

### 2.3. Association between Ultrasound and Clinical Symptoms

A significant association was observed between baseline VAS scores and SF (R = 0.416, *p* = 0.001) and SH (R = 0.394, *p* = 0.003), but not with color Doppler (R = −0.68, *p* = 0.63). Baseline WOMAC scores were significantly associated with SF (R = 0.346, *p* = 0.036), but not with SH (R = 0.195, *p* = 0.255) or color Doppler (R = −0.011, *p* = 0.96).

We also investigated whether real-time clinical improvement was associated with ultrasound-detected changes in synovitis. Spearman correlation analysis revealed that changes in SF were not associated with changes in VAS scores at the 1-month (R = 0.097, *p* = 0.448) or 6-month evaluation (R = 0.159, *p* = 0.213). Changes in color Doppler were negatively associated with changes in VAS scores at 1 month (R = −0.223, *p* = 0.008), but not at 6 months (ρ = 0.059, *p* = 0.063). Changes in synovial hypertrophy were not associated with changes in VAS scores at 1 (R = −1.58, *p* = 0.221) and 6 months (R = −0.1, *p* = 0.439).

Because the baseline KL grade can significantly affect HA therapy–associated outcomes, we performed a linear regression to determine whether the outcomes differed after adjustment for the KL grade. However, no significant association was found between changes in ultrasound parameters—color Doppler grade, SF, and SH—and VAS and WOMAC scores.

## 3. Discussion

In this study, we described the concurrent changes in ultrasound-detected synovitis and clinical outcomes of HA therapy for knee OA. Our findings indicated that HA therapy only non-significantly reduced imaging-detected synovitis and was not associated with clinical improvement. SF and SH correlated significantly with the baseline VAS and WOMAC scores, whereas the color Doppler grade did not change after HA therapy and was not correlated with clinical symptoms. These results imply that synovial imaging markers in knee OA may persist after short-duration HA therapy.

Knee pain originating from OA is multifactorial and has different presentations according to the deterioration of different structures or the development of neurological lesions [38]. A crucial source of ambulatory pain is knee instability, resulting from structural abnormality due to cartilage loss [8]. Another cause is the sensitization of synovial nociceptors resulting from progressive low-grade inflammation [39,40]. Cartilage loss is also associated with mild worsening of knee pain, and some knee pain is caused by worsening synovitis [8]. Together, these findings highlight how the synovium affects pain perception in patients with knee OA. Our study revealed that two consecutive HA injections were efficacious for both VAS reduction and WOMAC improvement; however, no association was noted with the changes in synovial parameters, suggesting that HA improved the VAS score, but not by significantly changing the synovial deficits detected through imaging. HA acts directly by desensitizing nociceptive nerve fibers [41], or indirectly by interacting with molecules such as intercellular adhesion molecule-1 (ICAM-1) and vascular cell adhesion molecule-1 (VCAM-1) in the synovial fluid to decrease inflammatory response and pain [42]. In our study, a significant association between imaging detection of synovitis and clinical symptoms was found only at baseline. Consistently, a recent study revealed that the arthroscopy-detected severity of knee synovitis and cartilage damage correlated with preoperative symptoms [43]. Although no significant association was found between the imaging detection of synovitis and clinical symptoms after HA therapy, the treatment exhibited good efficacy, with effects lasting up to 6 months. This may be partly due to the ultrasound-guided injection, which has been confirmed to be more accurate than blind injection [44]. In addition, ultrasound guidance can enable better fluid drainage than the blind procedure, thereby minimizing the dilution of HA in the synovial fluid and increasing its effectiveness.

The knee joint is a closed cavity, and the suprapatellar bursa is the largest bursa connected to the small bursa of the knee; thus, SF can serve as a good marker of the amount of fluid [36]. Our results revealed that despite two fluid drainage procedures and HA injections, only a mild decrease was found in the synovial fluid. Consistent with this observation, studies have demonstrated that the effect of joint lavage is temporary, and that fluid accumulation recurs within weeks [45,46] and, according to our data, can last up to 6 months. The nearly unchanged synovial fluid content indicates that it is a rather persistent condition that recurs even after complete fluid drainage. A study using steroid injections also reported similar persistence of the synovial fluid content, thus reinforcing that synovial fluid accumulation may be challenging to reverse [47]. It may result from the dysregulation of synovial cells as an inappropriate reaction to the mechanical stress response, whereas under normal circumstances, the cells secrete minimal fluid to supply nutrients to chondrocytes [7]. Normal synovial cell metabolism is destroyed under persistent inflammation. Future studies should explore the mechanisms underlying synovial cell sensors affected by mechanical fluid stress that cause them to secrete excessive fluid in knee OA.

Although OA is a low-grade inflammatory condition, the ultrasound-detected vascularity of an osteoarthritic joint varies in different stages and joints [6,48]. Our baseline evaluation revealed that the average color Doppler was 0.64, which was lower than grade 1 on a semiquantitative scale, suggesting that vascularity was low despite pain and functional disability. Similarly, a study also observed low vascularity in the suprapatellar bursa in patients with an OA knee [48]. Our results also revealed that HA therapy caused no significant change in color Doppler grading, implying that HA does not affect vascularity in synovitis. This finding is consistent with our previous study result demonstrating that HA therapy was associated with a non-significant change in color Doppler grading in both short-term and long-term evaluations in patients with RA ankle synovitis [21]. However, most of our patients had low-grade inflammatory synovitis, which may have made it challenging to quantify any changes through color Doppler examination. A study with a positive result reported a high baseline color Doppler value of 1.04, contrary to our value of 0.64, and that medium-molecular-weight HA therapy can result in a greater reduction in vascularity than steroid and high-molecular-weight HA in knee OA [49]. Another study with positive results suggested that HA therapy can reduce vascularity over the TMC joint; however, this study also had an initial high color Doppler value of 2.7 [50]. This might be because the TMC joint is smaller than the knee joint, with less effusion and synovial fluid.

This study has several limitations. First, ultrasound examination is not sufficient as a comprehensive evaluation of the synovial condition, especially when compared with MRI. However, our aim was to measure synovial effusion rather than to conduct a detailed pathological evaluation. The suprapatellar bursa has been considered to be one of the most representative ultrasound evaluation sites associated with clinical symptoms [51,52]. Second, the evaluation time was relatively short because we evaluated real-time changes in imaging parameters and clinical conditions. Third, we, only semiquantatively, evaluated the color Doppler findings and did not perform a thorough quantitative assessment. New techniques, such as the contrast-enhanced ultrasound used widely in RA synovitis, should be considered [53]. Fourth, this prospective cohort study lacked a control group because we primarily investigated the association of changes in synovial parameters with clinical symptom improvement. Future randomized control studies are warranted to validate our results.

## 4. Materials and Methods

### 4.1. Participants

In this prospective cohort study, we recruited patients with a symptomatic OA knee at a rehabilitation outpatient clinic in Taipei Veterans General Hospital’s Yuli branch. Our study was approved by the Ethics Committee of Taipei Veterans General Hospital (IRB No: 2017-03-009B; approval date: 9 August 2017). The inclusion criteria were as follows: (1) clinical and radiographic diagnosis of knee OA that met the American College of Rheumatology criteria [28,54,55], (2) age > 40 years, (3) disease duration > 1 month, (4) no active disease related to inflammation, (5) no previous knee surgery, and (6) no knee ankyloses or deformity. The exclusion criteria were as follows: (1) history of an allergic event after HA injection, (2) intra-articular infection in the past 3 months, (3) acute medical illness in the past 3 months, and (4) history of cancer, neurodegenerative disease, or other forms of dementia.

### 4.2. Drug Administration

**Figure 1:**
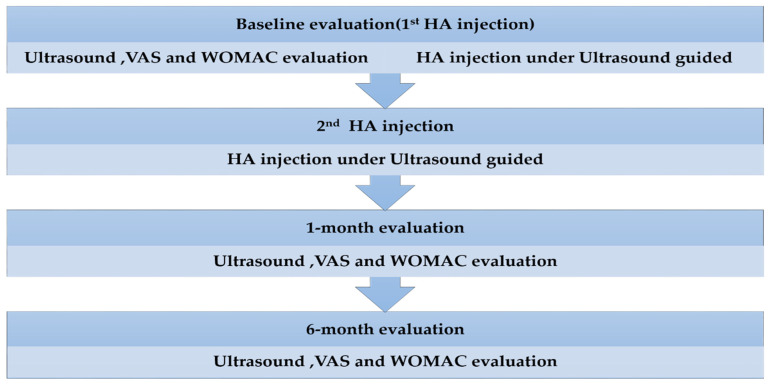
Study workflow. Two hyaluronic acid (HA) injections were administered with a 2-week interval, and post-treatment evaluations were performed at 1 and 6 months after the second injection. WOMAC, the Western Ontario and McMaster Universities Arthritis Index; VAS, visual analogue scale.

The study patients received two injections of Hyalgan (2.5 mL)—a low molecular-weight HA of 500–730 kDa (Bioibérica SA, Barcelona, Spain; molecular weight: 0.5 × 10^6^) [56]—with a 2-week interval between injections [21]. Clinical and ultrasound evaluations were performed at baseline and at 1 and 6 months after the second injection (Figure 1).

### 4.3. Ultrasound Evaluation

**Figure 2:**
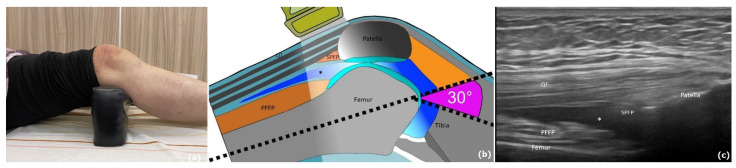
Ultrasound evaluation of the knee in a patient with stage II knee osteoarthritis and synovitis. (**a**) The patient lay supine with 30° knee flexion. (**b**) The ultrasound probe was placed over the knee just superior to the patella to examine the suprapatellar bursa. (**c**) Knee ultrasound longitudinal view indicating the position of the suprapatellar bursa between the patella and femur. PFFP, prefemoral fat pad; SPFP, suprapatellar fat pad; QT, trilaminar quadriceps tendon; Hypoechoic space (asterisk) reflects joint effusion.

**Figure 3:**
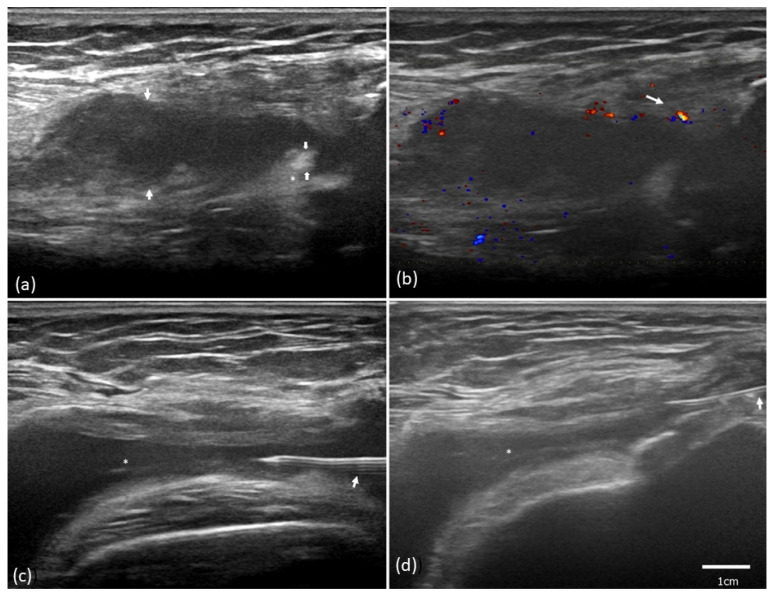
Ultrasound evaluation of synovitis and ultrasound-guided administration of hyaluronic acid (HA) in a patient with stage II knee osteoarthritis and synovitis. (**a**) The ultrasound revealed synovial hypertrophy (asterisk) and a synovial fluid depth of 1.34 cm in diameter (hypoechoic space between arrows). (**b**) Color flow imaging revealed grade 2 vascularity. (**c**) Ultrasound-guided procedure with a needle (arrow) for fluid (asterisk) aspiration. (**d**) The amount of fluid decreased and HA was injected with the needle (arrow).

Before the HA injection, baseline ultrasound examination was performed (Figure 2). Next, local anesthetic was injected subcutaneously. Fluid was drained completely under ultrasound guidance to prevent drug dilution. Subsequently, the HA injection was performed under ultrasound guidance (Figure 3c,d).

In accordance with the European Alliance of Associations for Rheumatology’s (EULAR’s) standardized procedures for rheumatic diseases [34], ultrasound examination was performed on the Aloka Prosound F75 Ultrasound System (Hitachi, Tokyo, Japan), equipped with a 6–18 linear array transducer, which is used for musculoskeletal evaluations. All ultrasound examinations were performed by the same physician, who had 10 years of experience in performing musculoskeletal ultrasounds, and under the same ultrasound settings. The patient lay supine with 30° knee flexion (Figure 2). Gray-scale imaging (B-mode) and the color Doppler ultrasound were used for evaluating structures and vascularity, respectively. The Doppler gain was adjusted after random noise was encountered by gradually decreasing the gain until the color noise disappeared near the cortical bone [57]. Both the longitudinal and transverse images were acquired for each sampled area of the knee. Synovial fluid depth (SF) in the suprapatellar bursa was defined as the distance between the highest and lowest points of the bursa (Figure 2). Synovial hypertrophy (SH) was quantified as the thickness of the synovial membrane in the suprapatellar bursa [58].

Knee vascularity was assessed using ultrasound color Doppler imaging (Figure 2b). The intraobserver reliability of color Doppler imaging has been reported previously [59]. A semiquantitative scoring system ranging from grades 0 to 3 was used to evaluate the amount of color in the visualized synovium in the knee joint [60], with grade 0 = no color pixels or flow in the visualized synovium; grade 1 = low flow or a single Doppler signal in the visualized synovium; grade 2 = color pixels in ≤50% of the visualized synovium; and grade 3 = color pixels in >50% of the visualized synovium [61]. Furthermore, we calculated the responder rate to evaluate the percentage of patients with a downgrading of the color Doppler scale at the 1-month and 6-month evaluations in comparison with the baseline values.

### 4.4. Clinical Outcome Measurement

**Figure 4:**
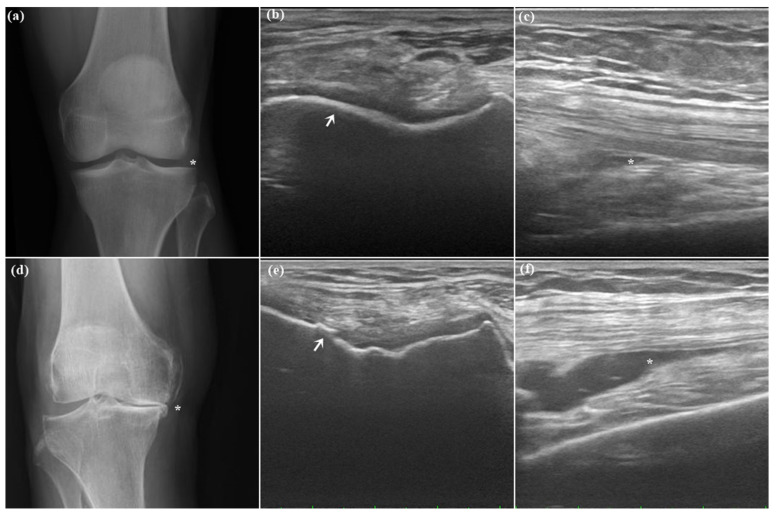
Knee X-ray (**a**,**d**) and ultrasound (**b**,**c**,**e**,**f**) in an individual with a healthy knee (**a**–**c**) and a patient with stage IV knee osteoarthritis (**d**–**f**). (**a**) Normal joint space without narrowing. (**b**) Normal hyaline cartilage (arrow). (**c**) Minimal joint effusion over SF (asterisk). (**d**) Large osteophytes, marked joint space narrowing, severe sclerosis, and definite bony deformity (asterisk). (**e**) Irregular hyaline cartilage (arrow). (**f**) Moderate effusion over SF (asterisk).

The baseline clinicodemographic characteristics included sex, age, body mass index (kg/m^2^), duration of knee pain, and medical history, including hyperuricemia or hyperglycemia. The radiographic severity of knee OA was assessed using KL grades, which were determined using anteroposterior knee X-ray films [62] (Figure 4).

Clinical variables included the global pain score measured using the visual analog scale (VAS) and knee function measured using the Western Ontario and McMaster Universities Osteoarthritis Index (WOMAC), and a validated self-administered questionnaire for measuring OA-related knee pain and disability [63]. The patients self-reported their daily average pain levels on the 100-mm VAS. The WOMAC comprises three subscales: functional knee pain (WOMAC_pain_, 5 items), physical function (WOMAC_phy_, 17 items), and stiffness (WOMAC_stiff_, 2 items). Each item is scored on a 0–4 scale, with total scores ranging from 0 to 96.

### 4.5. Data Analysis

SPSS (version 25.0; SPSS, IBM, Chicago, IL, USA) was used for all statistical analyses. The independent *t* test was used to assess continuous data, and the chi-square test was conducted to assess categorical data. The level of significance was set at *p* < 0.05. The baseline WOMAC, VAS, BMI, age, disease duration, SF, SH, and color flow image signals are reported as mean (95% confidence interval). The OA stage is reported as median (*interquartile range* [*IQR*]) (Table 1). The WOMAC, VAS, SF, and SH were assessed using the paired *t* test between the baseline and the 1- and 6-month evaluations.

Ordinal data, such as color Doppler values, were analyzed using nonparametric Mann–Whitney U and Kruskal–Wallis tests. Spearman’s test and linear regression were used to determine the association between synovitis and the measured outcomes—specifically, whether changes in VAS and WOMAC scores were correlated with changes in ultrasound parameters.

## 5. Conclusions

This study highlights the changes in ultrasound-detected synovial parameters after HA therapy for knee OA. Our data indicate that synovitis in knee OA, as measured using SF and SH, tends to persist and may be refractory to HA therapy despite clinical improvement and ultrasound-guided synovial fluid drainage before HA injection. Nevertheless, baseline SF and SH were correlated with VAS and WOMAC scores, suggesting that the severity of the synovial condition was closely related to symptoms. Moreover, low-grade vascularity was correlated with severity. Although the clinical efficacy of HA therapy was not improved by reducing synovial fluid or vascularity, our data provided a real-time synovial response to joint lavage with HA injection. Ultrasound-detected synovial parameters seem to be accurate for the real-time assessment of synovitis. Future studies should address different ultrasound-detected synovial responses to different therapeutic regimens in knee OA.

## Figures and Tables

**Table 1:** Baseline characteristics of the study population.

Characteristics	
*N*	103
Age (years), mean (95% CI)	70.09 (66.6–73.9)
M:F ratio, %	42:61
BMI (Kg/m2), mean (95% CI)	25.1 (22.9–27.2)
OA stage, median (IQR)	2 (1–4)
Disease duration (month), mean (95% CI)	22.22 (11.12–33)

Values are presented as means (95% CI) for continuous data and as medians (interquartile ranges) for noncontinuous data. WOMAC, the Western Ontario and McMaster Universities Arthritis Index; VAS, visual analog scale; BMI, body mass index.

**Table 2:** Ultrasound parameters and clinical evaluation at 1 and 6 months after treatment.

Characteristics	Baseline	1-Month Evaluation	6-Month Evaluation	*p* Value (1 Month to Baseline)	*p* Value (6 Months to Baseline)
Effusion	0.49 (0.43–0.56)	0.50 (0.43–0.57)	0.48 (0.42–0.54)	0.49	0.118
Synovial hypertrophy	0.34 (0.28–0.41)	0.35 (0.28–0.41)	0.38 (0.31–0.44)	0.918	0.446
Color flow Doppler	0.64 (0.50–0.78)	0.63 (0.49–0.77)	0.58 (0.44–0.72)	0.102	0.06
Global pain (VAS)	50.3 (40.3–55.3)	37.3 (26.8–47.7) *	36.0 (31.1–41.0) *	<0.001	<0.001
WOMAC	46.7 (42.6–50.8)	27.1 (22.9–31.1) *	42 (37.8–49.3)	<0.001	0.25

* *p* < 0.05 compared with the baseline. The values are presented as means (95% CI) for continuous data and as medians (interquartile ranges). WOMAC, Western Ontario and McMaster Universities Arthritis Index.

**Table 3:** Summary of color Doppler grading before and at 1 and 6 months after hyaluronic acid injection. The grading has been conducted in the entire study population.

Color Doppler Grade	0	1	2
Baseline evaluation	*n* = 39 (37.9%)	*n* = 61 (59.2%)	3 (2.9%)
1-month evaluation	*n* = 42 (40.8%)	*n* = 58 (56.3%)	3 (2.9%)
6-month evaluation	*n* = 47 (45.6%)	*n* = 53 (51.5%)	3 (2.9%)

## Data Availability

The data used to support the findings of this study are included within the article or are available from the corresponding author upon request.

## References

[1] Prieto-Alhambra D., Judge A., Javaid M.K., Cooper C., Diez-Perez A., Arden N.K. (2014). Incidence and risk factors for clinically diagnosed knee, hip and hand osteoarthritis: Influences of age, gender and osteoarthritis affecting other joints. Ann. Rheum. Dis..

[2] Cui A., Li H., Wang D., Zhong J., Chen Y., Lu H. (2020). Global, regional prevalence, incidence and risk factors of knee osteoarthritis in population-based studies. EClinicalMedicine.

[3] Hunter D.J., Schofield D., Callander E. (2014). The individual and socioeconomic impact of osteoarthritis. Nat. Rev. Rheumatol..

[4] Malanga G., Niazi F., Kidd V.D., Lau E., Kurtz S.M., Ong K.L., Concoff A.L. (2020). Knee Osteoarthritis Treatment Costs in the Medicare Patient Population. Am. Health Drug Benefits.

[5] Macchi V., Stocco E., Stecco C., Belluzzi E., Favero M., Porzionato A., De Caro R. (2018). The infrapatellar fat pad and the synovial membrane: An anatomo-functional unit. J. Anat..

[6] Robinson W.H., Lepus C.M., Wang Q., Raghu H., Mao R., Lindstrom T.M., Sokolove J. (2016). Low-grade inflammation as a key mediator of the pathogenesis of osteoarthritis. Nat. Rev. Rheumatol..

[7] Hunter D.J., Bierma-Zeinstra S. (2019). Osteoarthritis. Lancet.

[8] Bacon K., LaValley M.P., Jafarzadeh S.R., Felson D. (2020). Does cartilage loss cause pain in osteoarthritis and if so, how much?. Ann. Rheum. Dis..

[9] Wallace G., Cro S., Doré C., King L., Kluzek S., Price A., Roemer F., Guermazi A., Keen R., Arden N. (2017). Associations Between Clinical Evidence of Inflammation and Synovitis in Symptomatic Knee Osteoarthritis: A Cross-Sectional Substudy. Arthritis Care Res..

[10] Veronese N., Cooper C., Reginster J.Y., Hochberg M., Branco J., Bruyère O., Chapurlat R., Al-Daghri N., Dennison E., Herrero-Beaumont G. (2019). Type 2 diabetes mellitus and osteoarthritis. Semin. Arthritis Rheum..

[11] Courties A., Sellam J. (2016). Osteoarthritis and type 2 diabetes mellitus: What are the links?. Diabetes Res. Clin. Pract..

[12] Roman-Blas J.A., Castañeda S., Sánchez-Pernaute O., Largo R., Herrero-Beaumont G., Blanco F.J., Gómez R.B., Burlato M.C., González C.G., Vázquez J.L.G. (2017). Combined Treatment with Chondroitin Sulfate and Glucosamine Sulfate Shows No Superiority over Placebo for Reduction of Joint Pain and Functional Impairment in Patients with Knee Osteoarthritis: A Six-Month Multicenter, Randomized, Double-Blind, Placebo-Controlled Clinical Trial. Arthritis Rheumatol..

[13] Bruns C., Labisch S., Dirks J.H. (2022). 3D escape: An alternative paradigm for spatial orientation studies in insects. J. Comp. Physiol. A Neuroethol. Sens. Neural Behav. Physiol..

[14] Meng Z., Liu J., Zhou N. (2023). Efficacy and safety of the combination of glucosamine and chondroitin for knee osteoarthritis: A systematic review and meta-analysis. Arch. Orthop. Trauma Surg..

[15] da Costa B.R., Pereira T.V., Saadat P., Rudnicki M., Iskander S.M., Bodmer N.S., Bobos P., Gao L., Kiyomoto H.D., Montezuma T. (2021). Effectiveness and safety of non-steroidal anti-inflammatory drugs and opioid treatment for knee and hip osteoarthritis: Network meta-analysis. BMJ (Clin. Res. Ed.).

[16] Maheu E., Bannuru R.R., Herrero-Beaumont G., Allali F., Bard H., Migliore A. (2019). Why we should definitely include intra-articular hyaluronic acid as a therapeutic option in the management of knee osteoarthritis: Results of an extensive critical literature review. Semin. Arthritis Rheum..

[17] Ghosh P., Guidolin D. (2002). Potential mechanism of action of intra-articular hyaluronan therapy in osteoarthritis: Are the effects molecular weight dependent?. Semin. Arthritis Rheum..

[18] Lin W., Liu Z., Kampf N., Klein J. (2020). The Role of Hyaluronic Acid in Cartilage Boundary Lubrication. Cells.

[19] Iannitti T., Lodi D., Palmieri B. (2011). Intra-articular injections for the treatment of osteoarthritis: Focus on the clinical use of hyaluronic acid. Drugs RD.

[20] Gotoh S., Onaya J., Abe M., Miyazaki K., Hamai A., Horie K., Tokuyasu K. (1993). Effects of the molecular weight of hyaluronic acid and its action mechanisms on experimental joint pain in rats. Ann. Rheum. Dis..

[21] Wang C.C., Lee S.H., Lin H.Y., Liu F.W., Chiou H.J., Chan R.C., Chou C.L. (2017). Short-term effect of ultrasound-guided low-molecular-weight hyaluronic acid injection on clinical outcomes and imaging changes in patients with rheumatoid arthritis of the ankle and foot joints. A randomized controlled pilot trial. Mod. Rheumatol..

[22] Wang C.C., Wang C.T., Chou W.C., Kao C.L., Tsai K.L. (2020). Hyaluronic acid injection reduces inflammatory and apoptotic markers through modulation of AKT by repressing the oxidative status of neutrophils from osteoarthritic synovial fluid. Int. J. Biol. Macromol..

[23] Leighton R., Akermark C., Therrien R., Richardson J.B., Andersson M., Todman M.G., Arden N.K. (2014). NASHA hyaluronic acid vs. methylprednisolone for knee osteoarthritis: A prospective, multi-centre, randomized, non-inferiority trial. Osteoarthr. Cartil..

[24] Campbell K.A., Erickson B.J., Saltzman B.M., Mascarenhas R., Bach B.R., Cole B.J., Verma N.N. (2015). Is Local Viscosupplementation Injection Clinically Superior to Other Therapies in the Treatment of Osteoarthritis of the Knee: A Systematic Review of Overlapping Meta-analyses. Arthroscopy.

[25] Pasquali Ronchetti I., Guerra D., Taparelli F., Boraldi F., Bergamini G., Mori G., Zizzi F., Frizziero L. (2001). Morphological analysis of knee synovial membrane biopsies from a randomized controlled clinical study comparing the effects of sodium hyaluronate (Hyalgan) and methylprednisolone acetate (Depomedrol) in osteoarthritis. Rheumatology.

[26] Pereira T.V., Jüni P., Saadat P., Xing D., Yao L., Bobos P., Agarwal A., Hincapié C.A., da Costa B.R. (2022). Viscosupplementation for knee osteoarthritis: Systematic review and meta-analysis. BMJ (Clin. Res. Ed.).

[27] Zhao K., Wen Y., Bunpetch V., Lin J., Hu Y., Zhang X., Xie Y., Zhang S., Hongwei O. (2022). Hype or hope of hyaluronic acid for osteoarthritis: Integrated clinical evidence synthesis with multi-organ transcriptomics. J. Orthop. Transl..

[28] Kolasinski S.L., Neogi T., Hochberg M.C., Oatis C., Guyatt G., Block J., Callahan L., Copenhaver C., Dodge C., Felson D. (2020). 2019 American College of Rheumatology/Arthritis Foundation Guideline for the Management of Osteoarthritis of the Hand, Hip, and Knee. Arthritis Rheumatol..

[29] Jevsevar D.S., Brown G.A., Jones D.L., Matzkin E.G., Manner P.A., Mooar P., Schousboe J.T., Stovitz S., Sanders J.O., Bozic K.J. (2013). The American Academy of Orthopaedic Surgeons evidence-based guideline on: Treatment of osteoarthritis of the knee, 2nd edition. J. Bone Jt. Surg. Am..

[30] Johansen M., Bahrt H., Altman R.D., Bartels E.M., Juhl C.B., Bliddal H., Lund H., Christensen R. (2016). Exploring reasons for the observed inconsistent trial reports on intra-articular injections with hyaluronic acid in the treatment of osteoarthritis: Meta-regression analyses of randomized trials. Semin. Arthritis Rheum..

[31] Kohn M.D., Sassoon A.A., Fernando N.D. (2016). Classifications in Brief: Kellgren-Lawrence Classification of Osteoarthritis. Clin. Orthop. Relat. Res..

[32] Kaeley G.S., Bakewell C., Deodhar A. (2020). The importance of ultrasound in identifying and differentiating patients with early inflammatory arthritis: A narrative review. Arthritis Res. Ther..

[33] Hassan S. (2018). Overview of musculoskeletal ultrasound for the clinical rheumatologist. Clin. Exp. Rheumatol..

[34] Möller I., Janta I., Backhaus M., Ohrndorf S., Bong D.A., Martinoli C., Filippucci E., Sconfienza L.M., Terslev L., Damjanov N. (2017). The 2017 EULAR standardised procedures for ultrasound imaging in rheumatology. Ann. Rheum. Dis..

[35] Wang C.C., Wang C.T., Tsai K.L., Chou C.L., Chao J.K., Huang H.Y., Kao C.L. (2021). Effect of ultrasound-detected synovitis on therapeutic efficacy of hyaluronic acid injection for symptomatic knee osteoarthritis. Rheumatology.

[36] Kahle W., Frotscher M. (2002). Color Atlas and Textbook of Human Anatomy.

[37] Gomis A., Miralles A., Schmidt R.F., Belmonte C. (2009). Intra-articular injections of hyaluronan solutions of different elastoviscosity reduce nociceptive nerve activity in a model of osteoarthritic knee joint of the guinea pig. Osteoarthr. Cartil..

[38] Dray A., Read S.J. (2007). Arthritis and pain. Future targets to control osteoarthritis pain. Arthritis Res. Ther..

[39] Belluzzi E., Stocco E., Pozzuoli A., Granzotto M., Porzionato A., Vettor R., De Caro R., Ruggieri P., Ramonda R., Rossato M. (2019). Contribution of Infrapatellar Fat Pad and Synovial Membrane to Knee Osteoarthritis Pain. BioMed Res. Int..

[40] Eitner A., Pester J., Nietzsche S., Hofmann G.O., Schaible H.G. (2013). The innervation of synovium of human osteoarthritic joints in comparison with normal rat and sheep synovium. Osteoarthr. Cartil..

[41] Gomis A., Miralles A., Schmidt R.F., Belmonte C. (2007). Nociceptive nerve activity in an experimental model of knee joint osteoarthritis of the guinea pig: Effect of intra-articular hyaluronan application. Pain.

[42] Karatay S., Kiziltunc A., Yildirim K., Karanfil R.C., Senel K. (2004). Effects of different hyaluronic acid products on synovial fluid levels of intercellular adhesion molecule-1 and vascular cell adhesion molecule-1 in knee osteoarthritis. Ann. Clin. Lab. Sci..

[43] Olivotto E., Trisolino G., Belluzzi E., Lazzaro A., Strazzari A., Pozzuoli A., Cigolotti A., Ruggieri P., Evangelista A., Ometto F. (2022). Macroscopic Synovial Inflammation Correlates with Symptoms and Cartilage Lesions in Patients Undergoing Arthroscopic Partial Meniscectomy: A Clinical Study. J. Clin. Med..

[44] Fang W.H., Chen X.T., Vangsness C.T. (2021). Ultrasound-Guided Knee Injections Are More Accurate Than Blind Injections: A Systematic Review of Randomized Controlled Trials. Arthrosc. Sports Med. Rehabil..

[45] Reichenbach S., Rutjes A.W., Nüesch E., Trelle S., Jüni P. (2010). Joint lavage for osteoarthritis of the knee. Cochrane Database Syst. Rev..

[46] Paschos N.K., Giotis D., Abuhemoud K., Georgoulis A.D. (2014). Effectiveness of aspiration in knee joint effusion management: A prospective randomized controlled study. Knee Surgery Sports Traumatol. Arthrosc..

[47] Calvet J., Orellana C., Galisteo C., Garcia-Manrique M., Navarro N., Caixas A., Larrosa M., Gratacos J. (2018). Clinical and ultrasonographic features associated to response to intraarticular corticosteroid injection. A one year follow up prospective cohort study in knee osteoarthritis patient with joint effusion. PLoS ONE.

[48] Beitinger N., Ehrenstein B., Schreiner B., Fleck M., Grifka J., Lüring C., Hartung W. (2013). The value of colour Doppler sonography of the knee joint: A useful tool to discriminate inflammatory from non-inflammatory disease?. Rheumatology.

[49] Parisi S., Ditto M.C., Priora M., Borrelli R., Laganà A., Peroni C.L., Fusaro E. (2019). Ultrasound-guided intra-articular injection: Efficacy of hyaluronic acid compared to glucocorticoid in the treatment of knee osteoarthritis. Minerva Medica.

[50] Ingegnoli F., Soldi A., Meroni P.L. (2011). Power Doppler sonography and clinical monitoring for hyaluronic Acid treatment of rhizarthrosis: A pilot study. J. Hand Microsurg..

[51] Roemer F.W., Guermazi A., Felson D.T., Niu J., Nevitt M.C., Crema M.D., Lynch J.A., Lewis C.E., Torner J., Zhang Y. (2011). Presence of MRI-detected joint effusion and synovitis increases the risk of cartilage loss in knees without osteoarthritis at 30-month follow-up: The MOST study. Ann. Rheum. Dis..

[52] Crema M.D., Roemer F.W., Li L., Alexander R.C., Chessell I.P., Dudley A.D., Karlsten R., Rosen L.B., Guermazi A. (2017). Comparison between semiquantitative and quantitative methods for the assessment of knee synovitis in osteoarthritis using non-enhanced and gadolinium-enhanced MRI. Osteoarthr. Cartil..

[53] Liu H., Huang C., Chen S., Zheng Q., Ye Y., Ye Z., Lv G. (2019). Value of contrast-enhanced ultrasound for detection of synovial vascularity in experimental rheumatoid arthritis: An exploratory study. J. Int. Med. Res..

[54] Felson D.T., McAlindon T.E., Anderson J.J., Naimark A., Weissman B.W., Aliabadi P., Evans S., Levy D., LaValley M.P. (1997). Defining radiographic osteoarthritis for the whole knee. Osteoarthr. Cartil..

[55] Altman R., Asch E., Bloch D., Bole G., Borenstein D., Brandt K., Christy W., Cooke T.D., Greenwald R., Hochberg M. (1986). Development of criteria for the classification and reporting of osteoarthritis. Classification of osteoarthritis of the knee. Diagnostic and Therapeutic Criteria Committee of the American Rheumatism Association. Arthritis Rheum..

[56] Huang T.L., Chang C.C., Lee C.H., Chen S.C., Lai C.H., Tsai C.L. (2011). Intra-articular injections of sodium hyaluronate (Hyalgan^®^) in osteoarthritis of the knee. a randomized, controlled, double-blind, multicenter trial in the Asian population. BMC Musculoskelet. Disord..

[57] Torp-Pedersen S.T., Terslev L. (2008). Settings and artefacts relevant in colour/power Doppler ultrasound in rheumatology. Ann. Rheum. Dis..

[58] Sarmanova A., Hall M., Fernandes G.S., Valdes A.M., Walsh D.A., Doherty M., Zhang W. (2019). Thresholds of ultrasound synovial abnormalities for knee osteoarthritis—A cross sectional study in the general population. Osteoarthr. Cartil..

[59] Sarmanova A., Hall M., Fernandes G.S., Bhattacharya A., Valdes A.M., Walsh D.A., Doherty M., Zhang W. (2017). Association between ultrasound-detected synovitis and knee pain: A population-based case-control study with both cross-sectional and follow-up data. Arthritis Res. Ther..

[60] Torp-Pedersen S., Christensen R., Szkudlarek M., Ellegaard K., D′Agostino M.A., Iagnocco A., Naredo E., Balint P., Wakefield R.J., Torp-Pedersen A. (2015). Power and color Doppler ultrasound settings for inflammatory flow: Impact on scoring of disease activity in patients with rheumatoid arthritis. Arthritis Rheumatol..

[61] Szkudlarek M., Court-Payen M., Strandberg C., Klarlund M., Klausen T., Ostergaard M. (2001). Power Doppler ultrasonography for assessment of synovitis in the metacarpophalangeal joints of patients with rheumatoid arthritis: A comparison with dynamic magnetic resonance imaging. Arthritis Rheum.

[62] Kellgren J.H., Lawrence J.S. (1957). Radiological assessment of osteo-arthrosis. Ann. Rheum. Dis..

[63] Bellamy N., Buchanan W.W., Goldsmith C.H., Campbell J., Stitt L.W. (1988). Validation study of WOMAC: A health status instrument for measuring clinically important patient relevant outcomes to antirheumatic drug therapy in patients with osteoarthritis of the hip or knee. J. Rheumatol..

